# Oocyte Biobanks: Old Assumptions and New Challenges

**DOI:** 10.3390/biotech10010004

**Published:** 2021-02-18

**Authors:** Pamela Tozzo

**Affiliations:** Department of Molecular Medicine, Laboratory of Forensic Genetics, University of Padova, 35121 Padova, Italy; pamela.tozzo@unipd.it

**Keywords:** biobank, oocyte biobank, assisted reproductive technologies, “social freezing”

## Abstract

The preservation of fertility is a clinical issue that has been emerging considerably in recent decades, as the number of patients of childbearing age who risk becoming infertile for many reasons is increasing. The cryopreservation technique of oocytes has been developed for many years and nowadays constitutes a method of safe storage with impressive efficacy and high rates of successful thawing. The storage and use for research of oocytes taken for medical or non-medical can be carried out by both public and private structures, through egg sharing, voluntary egg donation and so-called “social freezing” for autologous use. This paper focuses on the oocyte bank as an emerging cryopreservation facility, in which a collaboration between public and private and the creation of a network of these biobanks can be useful in enhancing both their implementation and their functions. Good oocyte biobank practice would require that they be collected, stored, and used according to appropriate bioethical and bio-law criteria, collected and stored according to procedures that guarantee the best preservation of their structural components and a high level of safety, connected with appropriate procedures to protect the rights and privacy of the parties involved and associated with the results of the bio-molecular investigations that will be carried out gradually.

## 1. The Challenge of Female Fertility Preservation

The preservation of fertility is a clinical issue that has been emerging considerably in recent decades, as the number of patients of childbearing age who risk becoming infertile for many reasons is increasing.

One of the most relevant pathologies in this context is cancer [1]. Thanks to the early diagnosis of some cancers and the marked improvement in anticancer therapies, the survival rate of cancer patients has greatly increased. This means that women of childbearing age who have had cancer can hope, in the future, to conceive and become mothers. This has focused attention on the long-term consequences of the anticancer treatments to which they are subjected, and has raised the problem of maintaining gonadal function after healing [2]. The gonadotoxic effect of anticancer treatments—for example, chemo- and radiotherapy—on female reproductive potential has been known for a long time and can lead to partial or total suppression of fertility. The extent of gonadotoxic damage depends on various factors, such as the age of the patient, the type, grade, and stage of the tumor, the dosage and duration of chemo- and/or radiotherapy agent [3]. In women of childbearing age, anticancer treatments can compromise or interrupt ovarian function through a massive reduction in the number of follicles, thus inducing infertility and premature menopause [4]. On the other hand, in pre-pubertal girls, oncological treatments can lead to a subsequent absence of the menarche. To improve the lives of these patients, it is necessary to use cryopreservation techniques to preserve their fertility [5].

Cryobiology studies the effects of cryogenic temperatures on living matter and aims to cryopreserve the structural and physiological integrity of cells and tissues over time [6]. The cryopreservation of oocytes was impossible until recently, due to their great fragility. Oocytes are difficult cells to preserve and, although the first pregnancies from frozen oocytes were described in the late 1980s, for many years this technique underwent little improvement and little application until the vitrification technique was perfected. Today, with vitrification, oocytes can be successfully cryopreserved and can be used effectively even after a long time from harvest. Vitrification, in general, is the procedure for transforming a material into a glassy form, free from crystalline structure. In cryopreservation, a high concentration of cryoprotective agent is used simultaneously with the rapid drop in temperature. In this way, the oocyte is protected from any harmful actions due to the cryoprotective agent, while avoiding excessive dehydration and, above all, the creation of intracellular ice crystals [7].

The cryopreservation technique of embryos and oocytes with the vitrification method has been developed since 2007 and constitutes today a method of safe storage with impressive efficacy and high rates of successful thawing [8]. Cryogenic temperatures are achieved through the use of liquid nitrogen, inert, liquefied gas, at a temperature of −196.5 °C. The goal of cryobiology is to preserve cells and tissues that can subsequently be used for clinical and research applications [9]. The cryopreservation of ovarian tissue and gametes—oocytes and spermatozoa—are fundamental biomedical strategies for the preservation of fertility [10,11]. The application of these techniques has a total duration of about 2–3 weeks and requires an initial phase of ovarian stimulation through the administration of gonadotropins, drugs that induce multiple growth of the follicles. The cryopreservation of oocytes, embryos or blastocysts has a high efficiency from both a biological and clinical point of view and have no longer been considered experimental techniques since January 2013 [12]; with the vitrification technique, the procedure lasts a few minutes, without subjecting the embryo/oocyte to the more invasive and long method of cryopreservation, which had been practiced until recently [13].

However, there are limitations in the application of these techniques in prepubescent cancer patients and also in cancer patients of childbearing age who cannot undergo ovarian stimulation due to the need to immediately start radio/chemotherapy treatment, or due to the presence of a hormone-sensitive (e.g., breast cancer) tumor, since ovarian stimulation with estrogen may cause the primary tumor to worsen.

Oocyte cryopreservation does not present the ethical and legal criticalities of embryo freezing and has made it possible to preserve the potential of female fertility even in the absence of the partner. This method of preserving female fertility, initially conceived only for cancer patients of childbearing age, confident of becoming mothers after recovery, could in fact also be offered to women suffering from other diseases with a negative impact on fertility, or to healthy women, with the aim of procrastinating a pregnancy, in terms of egg freezing for non-clinical reasons.

A lively debate has been triggered on so-called “social egg freezing”, or on the possibility of using the cryopreservation technique by healthy and currently fertile women, who wish to postpone pregnancy for non-medical but social reasons—for example, for career reasons or because they have yet to find a reliable partner [14]. Different studies have reported that the choice to postpone motherhood is due to different factors related to lifestyle and societal changes, such as improved educational and professional opportunities for women, familial care commitments, economic difficulties, and need for greater financial security, absence of a partner, necessity to establish a stable home environment, improved access to contraception or a feeling of being “not ready” for parenthood [15,16].

It should be underlined that the age limits for becoming pregnant, even with Assisted Reproductive Technology (ART), do not change with cryopreservation. The success rates of ART remain around 25% under the age of 35, and only 10–15% after the age of 40 [17].

## 2. Oocyte Preservation

The storage and use of oocytes taken for clinical/therapeutic reasons or non-medical reasons can be carried out by both public and private institutions or other clinical organization, through egg sharing, voluntary egg donation, and so-called “social freezing” [18]. According to 2016 data from the Center for disease control and prevention, donated oocytes were used in about 9% of all assisted fertilization procedures with a doubling of the demand for donated oocytes between 2005 and 2016 [19].

With regard to egg sharing, this is a system for sharing eggs between a woman who performs a second-level homologous ART treatment and a couple who need other people’s eggs as they are not able to use their own [20]. Therefore, a woman who undergoes the ART proposes to donate to another couple who must undergo the same procedures. This material can be used afresh with fertilization of an embryo to be transferred immediately to the recipient already identified in advance, or cryopreserved for subsequent uses. The regulatory needs are evident: Alongside the action aimed at developing a culture of gratuitous and altruistic implementation of fundamental values of social solidarity, incentive practices could be envisaged [21].

When considering the voluntary donation of gametes, two essential issues should be addressed. On the one hand it would be useful to work for the spread of an authentic culture of altruistic and gratuitous donation in favor of other people; on the other hand, it is appropriate to discuss possible financial reimbursements of these donations and institution should responsibly assume the task of providing adequate compensation mechanisms for donors [22]. In other words, it means to operate within the regulatory framework that establishes the hinges of the system in the postulates of gratuitousness, voluntariness and altruism, providing, similarly to the hypotheses of blood and bone marrow donation, a system of expenses reimbursement that should be adequate to the specificity of the material and the significant psycho-physical or economic commitment of the donors. Learning from the experiences of other European countries subject to common EU legislation, a flat-rate reimbursement system could be hypothesized. To avoid any risk of commercialization and exploitation of the donor, a maximum reimbursement limit could also be envisaged, along with the maximum number of possible donations for the same donor. As far as so-called “social freezing” is concerned, this is a technological opportunity that involves decisive anthropological and social aspects [23,24]. In its essential features, it is a form of self-donation of gametes that is carried out at a young age to postpone the parenting project to a more mature age in which, as is known, one’s biological material will have reduced its reproductive potential [25,26]. In these cases, women decide to store their gametes in anticipation of possible illnesses or family/personal adverse events, or with a view to planning of one’s family and professional life postponing pregnancy to a more advanced age for educational or work reasons. This is a possibility offered by technologies for the preservation of one’s fertility as a preventive function of a future procreative difficulty connected with age or other non-medical reasons. Epidemiological studies have shown that women who opt for oocyte cryopreservation are commonly Caucasian, highly educated, middle-class professional women in their mid- to late 30s and the percentages of women who actually used their cryopreserved oocytes are very low, ranging from 6% to 12.1%. In this scenario, the possibility that the oocytes collected and stored in the context of so-called “social freezing” are eventually donated if they are not used by the donor can be seen in an altruistic perspective as a form of implementation of the donation system [15,27]. In fact, alongside the necessary dissemination and education action towards young people, which should also take place through the network of fertility and counselling centers, incentive practices could be envisaged such as the possibility of cryopreservation of biological material for free or at reduced cost if the donor agrees to donate part of the gametes to infertile couples who undergo heterologous ART treatment [14]. In this case, information is essential, in order to explain to these women that having cryopreserved gametes is not a fertility insurance, but may be the last tool to use when they find themselves (by age or by condition acute medical conditions) to have a significant reduction in their potential fertility. This may be a hypothesis to think about, given that it is necessary to prevent the risk of a disservice for these women who donate, in case they might think that their fertility is safeguarded by freezing eggs, which may cause them to postpone parenthood.

Once the feeding channels of oocyte donation have been specified, it is interesting to analyze how oocyte collections can be set up that have the structural and organizational characteristics of a biobank.

## 3. Oocyte Storage under a Biobanking Perspective

Biobanks perform a public function, although they are not necessarily established in public structures.

In complex pathologies, the success of research in the near future, more than what has happened in the past, will make use of the possibility of studying and characterizing biological samples of people suffering from various pathologies and undergoing therapeutic protocols. The number of samples required for biomolecular studies will always be higher and more difficult to obtain from a single structure: The establishment of networks of biobanks that supply continued access to the stored material is, therefore, of fundamental importance and it will allow for effective exchanges between clinicians and researchers [28].

The creation of this network will facilitate, through the definition of shared procedures, exchanges, and collaborations between the biobanks which are already operating, and the establishment of new units of service for health facilities that cannot or do not want to have their own biobank [29]. In fact, the establishment and maintenance of a biobank entail onerous investments: Coordination at a regional and national level is, thus, appropriate in order to drastically reduce these costs.

However, the transition from collections to harmonized biobanks is not simple from a technical point of view but, above all, from an ethical and legal point of view. If a sample is taken for a specific purpose, it is legitimate to ask whether and how it is possible to overcome the constraint and exploitation of the material in a different way.

The creation of biobanking networks also allows for the sharing of standards and procedures that guarantee the quality of the newly acquired preserved materials and the certification of the conservation status of those already present. In addition, from the standpoint of the information associated with the samples, the creation of networks and the consequent standardization enables the creation of and access to huge databases in which the data, homogeneously catalogued, can be found through common interfaces [30].

A biological sample taken for any reason in the present or in the past can become part, together with donor-related personal information, of a national or transnational network within which it becomes the object of scientific study, theoretically forever, and, furthermore, it may increasingly become the basis for products to be shared among institutions [31].

A research biobank may not directly carry out a research activity, but rather a service activity of researchers and citizens: It collects, stores, and distributes samples to the scientific community—research centers, universities, pharmaceutical and biotechnological industries—develop studies on the basis of what had been agreed to through the informed consent of each individual.

In this scenario, the biobank is fully improved and expresses its impartiality. It may be seen in an intermediate position between citizens, patients and researchers, guaranteeing the rights of all people involved [32].

The first reported pregnancy after the use of vitrified donor oocytes was made in 1999. Since January 2013, cryopreservation of oocytes is no longer considered an experimental technique, but an important strategy for the preservation of female fertility that has led to reproducible results. In the last 5–7 years, there has been a proliferation of biobanks that preserve human gametes, mainly for the purpose of providing gametes for heterologous fertilization pathways or to preserve their own gametes for future use. Alongside the need to establish standard institution and conservation procedures, discussions have also begun on the ethical issues that make this type of biobanks unique [33].

Regarding sample handling, good oocyte biobank practice would require that they be collected, stored, and used according to appropriate bioethical and bio-law criteria, and that they be collected and stored according to procedures that guarantee the best preservation of the structural components and a high level of safety (for instance, through storage in different refrigerators of aliquots of the same sample), connected with appropriate procedures to protect the rights and privacy of the parties involved and associated with the results of the bio-molecular investigations that will gradually be carried out.

Before proceeding to analyze the most critical aspects of an oocyte biobank, it may be useful to outline the main regulatory and ethical aspects underlying the creation of such a particular biobank as that of oocytes.

## 4. Oocyte Banking: Regulatory Framework and Ethical Concerns

Currently, egg banking can take place mainly in two contexts. The first is that of the collection and storage of oocytes by a woman for its future use. In turn, this type of banking can be implemented starting from two different assumptions: The woman may have acute and current medical reasons (for example because she is suffering from cancer and must undergo therapies that can compromise her reproductive capacity) or may choose to keep her eggs because she wants to postpone maternity (for example because she has not found a partner or for economic or career reasons). The second reason why an egg bank can be established is to collect donated eggs for future use by other women in the context of ART procedure. Finally, oocytes can also be collected for research purposes. The oocytes can be used for research after being collected in two ways: They can be oocytes donated for research or they can be oocytes taken for homologous or heterologous fertilization and not used.

As far as regulatory aspects are concerned, the European Commission has encouraged in the early 2000s the promotion of donation programs with the purpose to ensure the high quality and safety of tissue and cell donation. The European Commission considered it essential to encourage Member States to incorporate into their national legislation the principle of voluntary and unpaid donation. The Directive 2004/23/EC on Tissues & Cells seeks to ensure the quality and safety aspects of human tissues and cells used in therapies in the EU, and provides for a mechanism that will allow for a coherent approach to the authorization of imports and exports. Article 12 clearly states that donors may receive compensation, but this is strictly limited to making good the expenses and inconveniences related to the donation procedure. In this case, Member States define the conditions under which the compensation may be granted.

In Spring 2005, the European Commission carried out a survey among the Member States on the regulatory status of reproductive cell donation and the majority of European Member States reported legislation in respect of confidentiality, anonymity and non-remuneration for the gametes’ donor. What is surprising in this regard is that the importation and exportation of egg cells remains unregulated in the majority of countries [34].

A recent survey was performed by the European IVF Monitoring Consortium of the European Society of Human Reproduction and Embryology (ESHRE) on the legislation in EU about ART, sperm and eggs donation. The biggest recent change moving towards homogeneity in Europe is in the anonymity of egg and sperm donors. However, strict anonymity remains the law in 18 countries, including France, where regulatory developments are likely to change this requirement. In some countries anonymity applies to recipients but the born children can have access to donors’ identity when above a defined age (Austria, Croatia, Finland, Malta, Portugal, UK). In Germany and Switzerland, where anonymous donation is not allowed, recipients may bring their own donor to provide eggs just for that couple, a practice also allowed in all countries. Recent developments in direct-to-consumer DNA testing and the huge DNA databanks building up as a result means that anonymity can no longer be guaranteed anyway. Similarly, legislation has not caught up with egg freezing, which was made possible with the widespread introduction of fast-freezing by vitrification. However, the freezing of eggs (and sperm) for the preservation of fertility ahead of cancer treatment (i.e., for medical reasons) is allowed in all countries, despite an absence of specific legislation in 17 of them. Non-medical egg freezing is not permitted in Austria, France, Hungary, Lithuania, Malta, Norway, Serbia, and Slovenia, but is allowed in Germany and Switzerland [35].

Given that in the last 15 years there has been an improvement in harvesting and storage techniques, an increase in the demand for oocytes and a significant increase in the establishment of oocyte biobanks, both for clinical purposes (storage of own oocytes for medical or non-medical reasons, donation of oocytes for heterologous fertilization) or research.

A wide debate also on the ethical issues that arise in the field of egg donation. The ethical questions most discussed in the literature regarding oocyte banking for ART are essentially three, as highlighted by Kool and collaborators in a recent review on this topic [36]. First of all, the establishment of egg banks for heterologous fertilization is based on the voluntary donation by women who will have to undergo a medical procedure (to stimulate the production of oocytes and then to collect them) without there being any benefit for their health, so the interests of the donor and the potential child should be considered. Secondly, the improvement of vitrification techniques allows these oocytes to be preserved for a long time and this data can have repercussions on two fronts: On the one hand, it is necessary to improve information and integrate the consent of both the donor and the recipient and, on the other hand, it is necessary to better understand how long these oocytes can be used for heterologous fertilization or if they can, after a certain period of time, be discarded and used for research purposes. Finally, there is a large issue regarding the growth of demand and the scarcity of supply, for which questions may be raised about different of payment of oocyte donors, the fair distribution of oocytes available to recipients or the ethical acceptability of diverting part of these oocytes to the research removing them from clinical use. As stated by Kool and collaborators, there is a real gap in the literature on the further downstream aspects of egg retrieval from the donor, such as issues related to the storage, distribution, and use of these oocytes. For this reason, it is also advisable to analyze more general aspects of the establishment of oocyte biobanks that can pose new challenges for these sample collections.

The extent of the subject’s freedom of self-determination in relation to their own health is expressed in the principle of informed consent, through which the patient exercises their decision-making faculty in relation to the medical-surgical treatments which they intend or do not intend to undergo. The participation of a subject in a research through the use of a particular biological sample should always be a voluntary choice, validating the principle of donation. It is part of the principle of sociality, which commits every single person to realize themself in participating in the realization of the common good taking the form of an individual act, which contributes to the scientific progress of the community. The donation is a free, informed, and rescindable act and the possibility of withdrawing participation in a research is an expression of protection of individuals and their autonomy [31]. A question of particular importance involving biobanks and in particular the biological samples that constitute their soul, concerns the consent to participate in the biobanks themselves. One of the most important problems then consists in establishing within which limits and with what rules it is lawful to keep the samples beyond the time necessary to achieve the purpose for which the sample was collected and whether it is legitimate to use the same for purposes other than those initially identified. The justification of informed consent, in this particular area, pertains to the respect of the will of the donor to be informed about the intended use of the biological material and the information connected to it, and to guarantee the same the correctness of the procedures adopted for the protection of personal data. With more specific reference to the relationship between the biological sample and its possible uses, informed consent detects at least in three different moments. The first is identifiable after the subject has contacted the health facility, in the initial moment of sharing information with the doctor. The second is found in the sharing of data with other health professionals with insertion in the sample and information biobank. Finally, the third moment refers to the future and further sharing of that data with other subjects, who may be researchers outside the facility to which the sample has been donated.

The cryopreservation of oocytes for research purposes allows to reduce many of the legislative and ethical limits set by the cryopreservation of embryos for research, but it raises other ethical questions with respect to donation aimed at assisted fertilization techniques (whether homologous for medical or non-medical reasons or heterologous). The first question that arises is that of the risk of damage to which the woman is exposed in order to complete the collection procedure: It is a medicalization with different risks in order to obtain gametes without any benefit for the woman herself. Some authors have pointed out that the risk is excessively disproportionate to the benefit in this context [37]. However, in the name of the possibility of reimbursing the donor and guaranteeing information that is effectively exhaustive on the risks of the procedures, the possibility of recruiting egg donors with the sole purpose of using them for research has been accepted in various contexts [38].

## 5. The Challenges of Oocyte Biobanks: Governance Harmonization and Public Engagement

Biobanking could be compared to a great team game where everyone is organic and irreplaceable, with the citizen as activator and recipient of the process itself, the clinician who informs and collects the sample, the biobank which preserves and protects, and the researcher who opens up possibilities. Being informed and being made aware of the process and of the new scenarios which biobanking opens up is fundamental in order to guarantee citizen participation in biobanks [39]. If public information continues as a necessary environment for the implementation of a shared culture of biobanking and for conscious individual choices, then the motivation of healthcare professionals is also fundamental for an optimal collection of biospecimens and data, from ward staff to pathologists and to clinicians in charge of updating clinical information [40].

However, in order to unify the feeding channels and the uses of these biological samples, it is necessary to improve conservation techniques and find agreement on the timing beyond which the oocytes, no longer usable from a clinical point of view (and therefore for donation), can be used for research. In this way, one would shift from a bank of donated oocytes for clinical use to an oocyte bank used for research. Through the contribution of female donors/patients in terms of samples and information regarding their health and lifestyle, this type of biobank can become a precious resource for healthcare systems, also helping researchers to develop innovative approaches to personalized medicine, aimed at correlating lifestyle, environment and genes as determinants of women’s health, thus contributing to the development of adequate treatments and the prevention of diseases.

Although the goal of personalized medicine available to all in the short term is not realistic, some immediate benefits can be identified for female patients as a result of the development of an oocyte biobanking system and related activities. The availability of samples, for example, can provide more consistent data for molecular diagnostics and, in the short term, can represent an added value for basic research into reproductive medicine, allowing for more robust, replicable and precise standards for diagnosis, therapy and prognosis for fertility-related diseases. The creation of biobank networks can be useful because it may allow to have many more samples to use and therefore to be able to have reproducible results, to standardize procedures so that infrastructures can interact better both in the recruitment and in the use of samples, and to encourage efforts towards common governance. The importance of a standardized harmonization system in bio-banking is indirectly demonstrated by the fact that the ISO 20387:2018 (“Biobanking—General requirements for biobanking”) represents a reference standard for biobanks to adopt common strategies for the organization and processing of biological samples to achieve minimum standardization requirements [41,42].

Several immediate research benefits may follow the consolidation of a network of oocyte biobanks. Greater uniformity of governance and policy between public and private structures of oocyte biobanks can have several advantages [43]. To achieve the improvement and the uniformity of the techniques of conservation and the use of these oocytes, it is necessary to first clarify whether these oocytes will be used for donations or for research. In private oocyte banks, the market logic prevails with reference to the exchange, procurement, storage, and conservation of oocytes [44]. In these banks, moreover, the donors are selected with precise criteria, indicated on their websites, which mainly concern their age and state of health. From this point of view, one could think of a public–private partnership in which there is a convergence of oocytes donated spontaneously both for research and for reproduction and oocytes that are residual from unfinished ART procedures. The use could then be directed either to research or to donation for ART techniques (requested for health-related or social reasons). In this way, in public and private biobanks there could be the possibility of increasing the number of samples by converging research, clinical and reproductive needs [45].

The harmonization of standard operating procedures and best practices will lead to a higher level of efficiency for access to samples of guaranteed quality [46]. These quality samples will help raise the bar for biomedical and pharmaceutical research, thereby ensuring value for all stakeholders in the process, from academic and industrial researchers, to future patients and citizens [47]. The development of a supranational infrastructure and centralized databases can allow access to many more users, thus encouraging collaboration between researchers [48]. In this way, it will be possible to achieve the result of maximizing resources and sharing skills. The large number of samples available for collaborations will create much greater potential for translational research projects and facilitate effective and relevant basic research into reproductive medicine.

The ultimate aim that national or supranational authorities should set can be articulated in a plurality of actions aimed at informing and educating women as well as protecting the safety, health and privacy of all those involved in the procurement and use of donated oocytes [34]. In particular, the following issues should be addressed:

– Guaranteeing an adequate level of safety to protect the health of all subjects involved in the treatment of ART—donors and recipient couples in particular;
– preparing an information campaign through the healthcare system, upper secondary schools and universities and the world of work, aimed at education for the preservation of fertility and the spread of a culture of altruistic donation of gametes;
– regulating the essential aspects of the methods of gamete procurement and storage:
– egg-sharing: To address and regulate issues of incentive practices (fee exemption/reduction and placement of the donor on a priority list) and identification of gamete collection and storage centers;
– voluntary donation of oocytes: Identification of differentiated compensation mechanisms for donors and any further provisions on their status and donation methods to prevent marketing risks; and
– so-called “social freezing”: Identification of biobanks authorized to carry out cryopreservation and identification of any incentive practices to aid in the donation of biological material to benefit third parties (exemption/reduction of payment of cryopreservation costs).

An essential goal of an oocyte biobanking system, with public–private partnership, is also represented by the potential of economic return that this can generate [49]. The public–private partnership allows both an economic gain for the private component (which obviously also has economic gain among its objectives) and a reduction in costs for the public component as some costs can be substituted by the private counterpart. The knowledge economy requires that information be readily available, sufficiently robust and widely disseminated: A biobank is a highly rich source of relevant information of this type. The knowledge economy also requires a vibrant academic environment that, adequately supported, feeds the translation of results research into products. Research resulting from studies using materials from biobanks will certainly facilitate the development of state-of-the-art scientific knowledge and development of research capacity. An infrastructure of this value can also contribute to an increase in research and development in the industrial sector, with a strengthening of the levels of the economy, and a greater attraction of further investments in this path of recovery and research [50]. The research resulting from studies using materials from biobanks will certainly facilitate the development of scientific knowledge and the development of research skills. An infrastructure of this value may also contribute to an increase in research and development in the industrial sector, with a strengthening of the levels of the economy, and a greater attraction of further investments. Among the obvious long-term implications of an integrated approach to the management of a biobank network, are those resulting from an improvement in clinical and pre-clinical research quality standards which include, for example, a healthier workforce, new treatments and technologies with greater cost-effectiveness, savings to other parts of the healthcare system, and the marketing of products and technologies resulting in an increase in employment, tax revenues and exports. In this sense, the gain in economic terms can derive not only from an increase in the economic benefits for the territory hosting the biobank, but also in terms of reduction of health costs as more effective research creates more efficient health care, with reduction of care costs.

It would be necessary to use appropriate communication channels to educate women on three aspects: Responsible reproduction with an awareness of the physiological changes of one’s fertility, a greater culture of donation, both for clinical and research purposes and, finally, greater awareness of the possibilities, limits, and future prospects of research biobanks [51]. Biobanking activity presupposes the participation of citizens as donors as a fundamental fact [52]. In addition to the involvement of the individual patient/donor, it is important that in the general population there is a spread of the culture of biobanks: This means making sure that citizens know the main functions of a biobank and the importance of the possible donation of biological samples to a biobank. This assumption implies that, beyond the obvious need to illustrate the project to patients and to obtain their consent to the donation of their biomaterials, the general public should be informed about the nature of the biobanking activity and its purposes [53,54]. It is to be considered of paramount importance that all the subjects of the community be given the opportunity to know, at least in rough terms, the purposes of the biobanking activity and the guarantees offered to citizens by the biobank [55,56,57] and it is highly important to make the public aware of the objectives and methods of collecting and processing biomaterials and related data [58].

For this purpose, it is essential to provide citizens with correct information [59,60]. This can be done both through the means of communication (articles in newspapers, conferences, and websites), through the preparation of informative material aimed at the general population, and through patient associations. An important role also belongs to family doctors, who are duly informed and have the tools to answer any questions posed to them by patients. The activity of the biobank can also be presented in the context of voluntary associations in the health field, in order to promote the dissemination of the contents of the project to the population to encourage acceptance of the project. If public information, continuous and over time, is necessary environment for a shared culture of biobanking and for conscious individual choices, the motivation of health professionals is fundamental for an optimal collection of biomaterials and data, from ward staff to pathologists to clinicians in charge of updating clinical information.

## 6. Conclusions

Biobanks play a central role in biomedical research and, in particular, in the area of translational medicine, whose purpose is to close the gap between basic research (based on the study of molecules, cells, and tissues) and research applied to the clinical world which actually reaches the patient’s bed. To fully realize the promises of personalized medicine, whose objective is to offer each patient the best possible care, it is, in fact, necessary to have access to large numbers of samples and quality data.

Recently, attention has increased towards oocyte biobanks in which gametes are collected for different reasons and can be used for different purposes. The creation and implementation of networks of these biobanks in which a true collaboration between public and private can be implemented is a promising scenario that can lead to great benefits for research and clinical practice in the field of human reproduction. Oocyte biobanks may be seen as visionary institutions: Their foundation sees its purposes in view of future projects and studies, thus allowing for an improvement in the quality of research. All of this is in the furtherance of the ultimate aims of medicine: to study, to cure and to prevent disease.

## Data Availability

Data sharing not applicable.
